# Interactions between Natural Populations of Human and Rodent Schistosomes in the Lake Victoria Region of Kenya: A Molecular Epidemiological Approach

**DOI:** 10.1371/journal.pntd.0000222

**Published:** 2008-04-16

**Authors:** Michelle L. Steinauer, Ibrahim N. Mwangi, Geoffrey M. Maina, Joseph M. Kinuthia, Martin W. Mutuku, Eric L. Agola, Ben Mungai, Gerald M. Mkoji, Eric S. Loker

**Affiliations:** 1 Department of Biology, University of New Mexico, Albuquerque, New Mexico, United States of America; 2 Centre for Biotechnology Research and Development, Kenya Medical Research Institute, Nairobi, Kenya; Biomedical Research Institute, United States of America

## Abstract

**Background:**

*Schistosoma mansoni* exists in a complex environmental milieu that may select for significant evolutionary changes in this species. In Kenya, the sympatric distribution of *S. mansoni* with *S. rodhaini* potentially influences the epidemiology, ecology, and evolutionary biology of both species, because they infect the same species of snail and mammalian hosts and are capable of hybridization.

**Methodology/Principal Findings:**

Over a 2-year period, using a molecular epidemiological approach, we examined spatial and temporal distributions, and the overlap of these schistosomes within snails, in natural settings in Kenya. Both species had spatially and temporally patchy distributions, although *S. mansoni* was eight times more common than *S. rodhaini*. Both species were overdispersed within snails, and most snails (85.2% for *S. mansoni* and 91.7% for *S. rodhaini*) only harbored one schistosome genotype. Over time, half of snails infected with multiple genotypes showed a replacement pattern in which an initially dominant genotype was less represented in later replicates. The other half showed a consistent pattern over time; however, the ratio of each genotype was skewed. Profiles of circadian emergence of cercariae revealed that *S. rodhaini* emerges throughout the 24-hour cycle, with peak emergence before sunrise and sometimes immediately after sunset, which differs from previous reports of a single nocturnal peak immediately after sunset. Peak emergence for *S. mansoni* cercariae occurred as light became most intense and overlapped temporally with *S. rodhaini*. Comparison of schistosome communities within snails against a null model indicated that the community was structured and that coinfections were more common than expected by chance. In mixed infections, cercarial emergence over 24 hours remained similar to single species infections, again with *S. rodhaini* and *S. mansoni* cercarial emergence profiles overlapping substantially.

**Conclusions/Significance:**

The data from this study indicate a lack of obvious spatial or temporal isolating mechanisms to prevent hybridization, raising the intriguing question of how the two species retain their separate identities.

## Author Summary

One of the world's most prevalent neglected diseases is schistosomiasis, which infects approximately 200 million people worldwide. *Schistosoma mansoni* is transmitted to humans by skin penetration by free-living larvae that develop in freshwater snails. The origin of this species is East Africa, where it coexists with its sister species, *S. rodhaini*. Interactions between these species potentially influence their epidemiology, ecology, and evolutionary biology, because they infect the same species of hosts and can hybridize. Over two years, we examined their distribution in Kenya to determine their degree of overlap geographically, within snail hosts, and in the water column as infective stages. Both species were spatially and temporally patchy, although *S. mansoni* was eight times more common than *S. rodhaini*. Both species overlap in the time of day they were present in the water column, which increases the potential for the species to coinfect the same host and interbreed. Peak infective time for *S. mansoni* was midday and dawn and dusk for *S. rodhaini*. Three snails were coinfected, which was more common than expected by chance. These findings indicate a lack of obvious isolating mechanisms to prevent hybridization, raising the intriguing question of how the two species retain separate identities.

## Introduction

One of the world's most prevalent neglected diseases is schistosomiasis, which is caused by flatworms of the genus *Schistosoma*. It is estimated that 200 million people world wide are infected [1]. Schistosomiasis is notable for its chronic nature, for being difficult to control on a sustained basis, and for the limited options currently available for control [2]. *Schistosoma mansoni* is the most widespread and best known of the human-infecting schistosomes. It is a genetically diverse parasite with complex epidemiology, particularly in East Africa, which is also its hypothesized place of origin [3].

Epidemiological studies of *S. mansoni* understandably often focus on human infections [4], but due to the longevity of schistosome infections in the human host and to the high vagility of humans, studies of humans alone make it difficult to detect when and where transmission actually occurs. By examining snails, the obligatory hosts for the larval stages of schistosomes, we can gain a much needed perspective, one that allows the determination of where human-infective cercariae are actually being produced, and thus identifies likely sites of active transmission. Also, during the molluscan phase of the schistosome life cycle, schistosome sporocysts may encounter other individuals of the same or a related schistosome species, or of unrelated species of digenetic trematodes (see [5] for an overview of some of the possible interactions), potentially influencing the dynamics of transmission. Molecular epidemiological investigations have shown that *S. mansoni* infections tend to be overdispersed (aggregated in a small proportion of host individuals) in their molluscan hosts, with some snails harboring as many as 9 distinct parasite genotypes [6],[7]. Such patterns could be the result of differing levels of susceptibility, acquired immunity [7],[8], microhabitat variation of snails and miracidia, and/or competitive interactions within the snail, and as they may influence transmission of infection to humans, should be further investigated.

In western Kenya, where our studies were undertaken, *S. mansoni* is likely to encounter and interact with its sister species, *S. rodhaini*. This species is typically considered a parasite of rodents although it has been reported from wild felids, canids, and even humans, although this latter observation has not been confirmed with molecular techniques [9]–[14]. Evidence from experimental infections of baboons suggests *S. rodhaini* cannot infect these primates unless they are coinfected with *S. mansoni* [15]. Although *S. mansoni* is primarily a parasite of humans and secondarily other primates, rodents can serve as reservoir hosts, including in East Africa [16]. In some locations such as Guadeloupe, rodents are the exclusive definitive host for *S. mansoni* [17]. Overlap of both schistosome species in the same individual rodent host was reported by Schwetz [18] who found eggs of both species in rodents the Democratic Republic of the Congo, although he considered the eggs shaped like those of *S. mansoni* to be a different variety of this species. Both schistosome species infect the same species of *Biomphalaria* snails and past reports indicate that they can infect the same individual snail host [19]; therefore they potentially influence each other in terms of infection patterns, development, and cercarial release patterns. Also, these two species hybridize readily in the laboratory [20]–[22] and a natural hybrid has been found from a snail in the Lake Victoria region [23].

Hybridization is an important epidemiological concern because hybrids could directly infect humans or lead to gene introgression between the species, which both could alter their biology and capacity to cause pathology. However, in the face of possible hybridization and definitive and intermediate host overlap, these two species are apparently able to maintain their identity [23], which unless contact is very recent, suggests the presence of isolating mechanisms including ecological, geographical, or temporal isolation. Théron and Combes [24] hypothesized that the time of day of cercarial emergence of each species could serve as an isolating mechanism since at different times of the day, different host species would be utilizing aquatic habitats. Most schistosome cercariae emerge from their snail hosts following a predictable circadian pattern [25]–[27], one that is genetically controlled [28]. *Schistosoma mansoni* cercariae are diurnal and are typically released during daylight hours, but populations vary concerning their exact time of emergence ([17] and references therein). Previous studies have shown that *S. rodhaini* is nocturnal and emerges after dark between 18:00–22:00 hours [27],[29]. These emergence times correspond to times when their putative hosts are present in the water and available for infection, humans during the day and rodents at night. However, schistosome cercariae remain active and infective in the water column for up to 9 hours in an experimental setting [30]. This longevity creates the potential for overlap in actual transmission times, even if the cercariae emerge at different times.

Using schistosome specimens derived from field collections of snails over a two year period in the Lake Victoria region of Kenya, and applying molecular techniques to these specimens, we addressed several questions concerning the epidemiology of *S. mansoni* and *S. rodhaini*, and investigated potential ecological, spatial, and temporal isolating mechanisms: 1. Do *S. mansoni* and *S. rodhaini* co-occur spatially and temporally and how prevalent are they? 2. Does either species outnumber the other in terms of number of snails infected and number of cercariae produced per snail? 3. How common are hybrids in snails? 4. How are both species distributed within their snail hosts in terms of abundance (number of genotypes per snail), and how does this correspond to the number of cercariae produced? 5. Can snails become coinfected with both species and is there any evidence the two species co-occur more or less often than expected by chance? 6. Do these species overlap on a microtemporal scale, or is there overlap in the circadian pattern of cercarial emergence for each species? 7. How are these patterns influenced when snails are coinfected with multiple multilocus genotypes or species?

## Methods

### Snail Monitoring and Infections of Mice

Snails were collected at various sites in western Kenya in the Lake Victoria Basin (Table 1). Snails were isolated in individual wells of tissue culture plates in aged tap water for 24–48 hours and examined for shedding cercariae. Infected snails were given an individual identification number and their cercariae were used to infect mice (Swiss Albino, male and female, 6–7 weeks old), in most cases two mice per infected snail. Infections were performed via skin penetration of the abdomen while the mice were anesthetized with sodium pentobarbital. Infection doses of 10 to 200 cercariae were used depending on the number released by the snail.

**Table 1:** Collection sites for snails of the genus *Biomphalaria* and their schistosome parasites in Western Kenya.

Site Name	Habitat Type	South	East	Infection
Nawa	Lake Channel	−0.1019444	34.7133333	Present
Asembo Bay	Lake Shore	−0.1885080	34.3875340	Present
Car Wash	Lake Shore	−0.0958667	34.7485944	Present
Fisheries Station	Lake Shore	−0.0892150	34.7393400	Absent
Hippo Point	Lake Shore	−0.1250020	34.7418030	Absent
Homa Bay	Lake Shore	−0.5226060	34.4545590	Present
Kagwel	Lake Shore	−0.1911111	34.5033333	Present
Kaugenge	Lake Shore	−0.4638889	34.2783333	Absent
Kisuwi Beach	Lake Shore	−0.4397222	34.2336111	Absent
Lambwe Beach	Lake Shore	−0.4363100	34.2502420	Absent
Lela Beach	Lake Shore	−0.5147222	34.4744444	Absent
Mbita Beach	Lake Shore	−0.4213889	34.2075000	Absent
Powerhouse	Lake Shore	−0.0922833	34.7525694	Present
Sandharvester Site	Lake Shore	−0.1013889	34.7147222	Present
Seka Kagwa	Lake Shore	−0.3555556	34.6827778	Present
Tilapia Beach	Lake Shore	−0.0933333	34.7608333	Present
Usare Beach	Lake Shore	−0.1057120	34.6742900	Present
Nyabera	Marsh	−0.1097139	34.7746111	Present
Asao	Stream	−0.3325600	34.9991440	Present
Kasabong	Stream	−0.1519060	34.4455280	Present
Lwanda	Stream	−0.4769444	34.2888889	Present
Sigalagala	Stream	−0.1284500	34.7476410	Absent

Global Positioning System coordinates are projected in North American Datum 1983 decimal degrees.

Infected snails were subjected to 24 hour cercarial release profiles every 4–7 days after collection for as long as they survived. Profiles were created by counting the number of cercariae released every hour for 24 hours as the snails were moved hourly between wells of 24 well tissue culture plates, each well with 1 mL of aged tap water. Snails were kept under natural lighting (not direct sunlight) in Kenya in a laboratory with east facing windows. Additional replicates were performed in a laboratory with west facing windows and the peak emergence times did not change. Cercariae were either counted directly using a stereomicroscope if few were released, or a subsample was counted by mixing the well with a pipette, removing a subsample of 200 µL, and counting them on a gridded plate after staining with iodine. The final count was then multiplied by 5 to estimate the number in 1 mL. To determine if snails were shedding multiple genotypes or multiple species at different time intervals, cercariae were pooled into 4 time intervals (3:00–9:00, 9:00–15:00, 15:00–21:00, and 21:00–3:00) and used to infect 1–2 mice per time interval. Recovery of adult worms from mice 7 weeks post-exposure was accomplished by perfusion [31]. Gender of the worms was determined by examining adult morphology and was generally obvious with a few exceptions of infections with immature worms, which were scored as unknown. Adult worms were stored in 95% ethanol at 4°C until further use. The methodology described above has been fully approved for the use of animals by the University of New Mexico Institutional Animal Care and Use Committee (Protocol #07UNM003) and Board of Animal Care and Use of the Kenya Medical Institute.

### Molecular Methods

Adult worms recovered from the mice were subsampled so that at least 16 individuals from every snail during each time interval were assayed if available. Snails did not shed during all time intervals and not all infections yielded at least 16 worms. The HotSHOT [32] method was used to prepare genomic DNA of the worms for PCR. To determine the number of genotypes of cercariae that were released from a snail, 7 previously published microsatellite loci [33],[34] were amplified in 1 multiplexed PCR reaction, the P17 panel, as described by Steinauer et al [35]. PCR products were genotyped using an ABI3100 automated sequencer (Applied Biosystems) and scored with GeneMapper® v. 4.0 (Applied Biosystems) software. All genotype calls were verified manually. Individuals with the same genotypes at all 7 loci that emerged from the same snail were considered to be clones descended from a single miracidium and are referred to as a multilocus genotype, although the probability that identical individuals arose from sexual reproduction was also calculated with GENCLONE 1.1 [36]. Part of the 16S and 12S genes (16S-12S) of the mitochondrial DNA from each multilocus genotype was amplified and sequenced using the method of Morgan et al. [23]. Sequences were submitted to GenBank Data Libraries (Accession numbers EU513397-EU513598)

Both the 16S-12S data and microsatellite data were used for species identification. Reference individuals from laboratory reared specimens and also field collected specimens of *S. mansoni* from Kenya, Egypt, and Brazil were used to establish species level differences with the markers. The 16S-12S data was aligned along with reference sequences from GenBank (*S. mansoni*: AY446260 and AY446261 (Madagascar); AY446262 and AY446263 (Kenya); AY446259 (Ghana), AF531310 (Tanzania); and *S. rodhaini*: AF531309, AY446265, and AY446264 (Kenya). The total dataset included the following number of specimens for each species: *S. mansoni*, 190; *S. rodhaini*, 24; *S. haematobium*, 1; *S. bovis*, 2. Sequences were aligned with ClustalX [37] using a gap opening penalty of 15 and extension penalty of 0.2. Identical sequences were identified using Sequencher 4.6 (Genecodes) and redundant sequences were removed from the alignment. Phylogenetic analyses using the minimum evolution optimality criterion was performed on the data using the model of evolution selected by the likelihood ratio test implemented in MODELTEST 3.0 [38]. Tree searches were done heuristically using PAUP* 4.0b10 [39] with tree bisection reconnection (TBR) branch swapping on initial trees that were obtained by random stepwise addition of taxa, replicated 100 times. Node support for the node separating *S. mansoni* and *S. rodhaini* was assessed by bootstrap analysis [40] using the faststep option with 10,000 pseudoreplicates. Species identification was based on clustering with reference sequences from GenBank. Genetic divergence was calculated using MEGA version 2.1 [41]. Within clade divergences and net between clade divergences were calculated using uncorrected p-distances, which is the proportion of sites that differ between two taxa. For the microsatellite data, a population assignment test was performed with GenAlEx [42] using the “leave one out method” to assess whether the microsatellite markers agreed with the 16S-12S data and could differentiate the species using the 7 microsatellite loci. The loci were also compared by eye to determine which were able to differentiate the species.

### Data Analysis

Prevalence, or percentage of infected snails, of schistosomes and of each schistosome species was calculated for each collection and also pooled across collections by site (Table 2). A proportion of infections (33%) could not be identified to species because the snails never released enough cercariae to infect mice, the mice did not become infected by the cercariae, or the mice died before worms could be recovered. Therefore, estimated prevalence values were also calculated by apportioning the total prevalence value to each species based on their proportion in the known specimens at each site. Both raw prevalence and estimated prevalence values are given in Table 2. To test if prevalence (raw values) and mean intensity (number of genotypes per snail) of infection was positively correlated as noted in previous studies [43], a Pearson's correlation was calculated on the log transformed values using the same software. Also, an analysis of covariance (ANCOVA) that examined the difference in the total number of cercariae released between species and its relationship to snail size was performed. Only snails infected with a single genotype and that shed more than 90 cercariae were used in this analysis. The model included species as a categorical variable and snail size as a covariable as well as the interaction between the terms.

**Table 2:** The number of snails infected with *Schistosoma mansoni* (SM), *Schistosoma rodhaini* (SR), unidentified mammalian schistosomes (US), and total schistosomes (TS) at various sites in the Lake Victoria Basin of Kenya.

Site	Snails Infected With				Total Snails	P	SM P	SM P*	SR P	SR P*
	SM	SR	US	TS						
Kagwel	1	0	0	1	934	0.11	0.11	0.11	0	0
Lwanda	1	1	0	2	923	0.22	0.11	0.11	0.11	0.11
Sandharvest	12	0	5	17	2652	0.64	0.45	0.64	0	0
Nyabera	8	3	2	13	2066	0.65	0.39	0.47	0.15	0.18
Seka Kagwa	5	0	1	6	747	0.80	0.67	0.67	0	0
Homa Bay	4	0	9	13	1605	0.81	0.25	0.81	0	0
Powerhouse	3	0	2	5	544	0.92	0.55	0.92	0	0
Nawa	5	5	2	12	1166	1.03	0.43	0.52	0.43	0.52
Asao	44	1	19	63	5997	1.05	0.73	1.03	0.02	0.02
Asembo Bay	21	2	9	31	2723	1.14	0.77	1.04	0.07	0.10
Usare Beach	1	1	6	10	816	1.23	0.12	0.62	0.12	0.62
Tilapia Beach	5	0	11	16	1169	1.37	0.43	1.37	0	0
Kasabong	9	0	7	16	450	3.56	2.00	3.56	0	0
Car Wash	22	4	5	31	849	3.65	2.59	3.09	0.47	0.56
**Total**	**141**	**17**	**78**	**236**	**22641**	**1.04**	**0.62**	**0.92**	**0.08**	**0.11**

The total number of snails collected, prevalence of schistosome infection (P), and prevalence of confirmed *S. mansoni* (SM P) and *S. rodhaini* (SR P) infections are given for each site. Prevalence for each species was also adjusted as denoted by an asterisk to accommodate unidentified schistosomes by multiplying the prevalence of schistosomes by the percentage of identified schistosomes for each species.

To determine if coinfections in snails were random occurrences or if they were the product of a structured community, the observed parasite communities were compared to a null models of communities based on the observed values of the species' prevalence as described by Lafferty et al. [44]. Expected numbers of coinfected snails were calculated as the product of the number of snails collected and the prevalence (as a proportion) of each parasite species present in the population at the site of interest, during the time of interest (not pooled spatially or temporally). The expected number was compared to the observed number using χ^2^ goodness of fit tests.

Two-tailed Fisher's Exact tests were used to detect if the proportion of each genotype of cercariae shed from multiply infected snails varied among replicates over time using VassarStats (www.faculty.vassar.edu/lowry/VassarStats.html). Only one snail yielded enough data to examine the three way relationship among genotype, replicate, and time of day (most snails yielded adults mostly from a single time period, 9:00–15:00). This snail was coinfected with both schistosome species, 3 genotypes of *S. rodhaini* and 1 genotype of *S. mansoni*. These data were analyzed with a 3-way contingency table with a log-linear analysis for goodness of fit using VassarStats, and the standardized deviates were examined to determine which categories contributed the most to observed significant values.

## Results

### Species Identification

**Figure 1:**
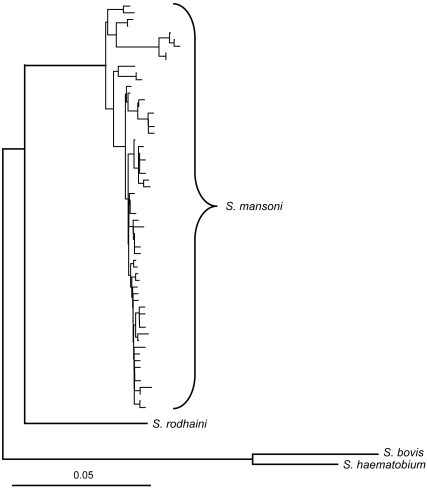
Minimum evolution tree of several specimens of *Schistosoma mansoni* and *S. rodhaini* based on 512 bp of mitochondrial DNA including part of the 16S ribosomal RNA gene, all of thetRNA-Cys gene, and part of the 12S ribosomal RNA gene. Uncorrected p-distance is given for scale.

**Figure 2:**
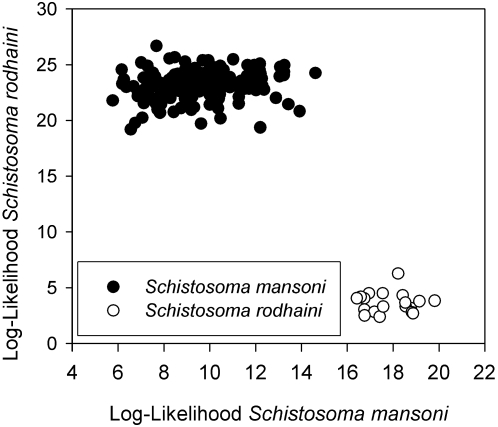
Population assignment graph of *S. mansoni* and *S. rodhaini* based on 7 microsatellite markers. The dot plot indicates the log-likelihood (absolute value) that each individual belongs to either species. The lower the log likelihood value, the more likely the individual belongs to that species.

Alignment and removal of redundant sequences yielded 512 bp for 64 taxa: 61 *S. mansoni* and one each of *S. rodhaini, S. bovis, and S. haematobium*. The evolutionary model selected by the likelihood ratio test implemented by MODELTEST 3.0 [38] was the unequal-frequency Kimura 3-parameter model. Phylogenetic analysis yielded 9 trees that did not differ in their groupings of specimens between species (Fig. 1). Within *S. mansoni* 1.5% sequence divergence was detected; however, no variation was detected in *S. rodhaini* (24 specimens) or *S. bovis* (2 specimens). The net between groups genetic distance between *S. mansoni* and *S. rodhaini* was 9.3%, which was greater than the distance between S. *haematobium* and *S. bovis* (7.6%). A population assignment test using the microsatellite markers yielded 100% assignment of the individuals of *S. mansoni* and *S. rodhaini* to their species based on the 16S-12S data (Fig. 2). Two loci were completely non-overlapping between *S. mansoni* and *S. rodhaini* (SMD28 and SMD89 from [34]), and one locus (SMD43 from [33]) did not amplify in *S. rodhaini*. There was no evidence of hybrids based on the mtDNA and microsatellite markers which were concordant in their identification of each individual. Also, no individuals were found to have microsatellite signatures that were indicative of hybrids either in the nonoverlapping loci or the other loci as shown by the population assignment test, which placed the species in relatively tight groups (Fig. 2).

### Snail Collections and Parasite Distribution

A total of 22,641 snails were collected in the Lake Victoria basin over a 2 year period. Of these snails, 236 (157 *B. sudanica* and 79 *B. pfeifferi*) were infected with schistosomes, a prevalence of 1.04%. Not all schistosome infections were identified, but of the 167 that were, 90% were *S. mansoni* and 8.1% were *S. rodhaini*, and 1.9% were mixed species infections. Most infections of *S. rodhaini* occurred in *B. sudanica* and only one individual of *B. pfeifferi* was infected with this species, which was a coinfection with *S. mansoni*. The sex ratio of adults obtained from mice of *S. mansoni* was male biased (2.36), while that of *S. rodhaini* was more equivalent (1.11).

**Figure 3:**
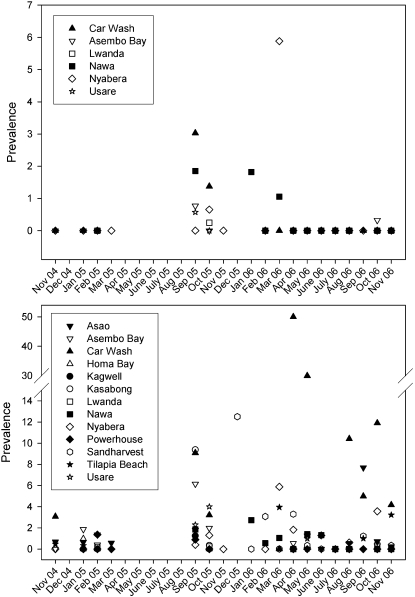
Prevalence (percent of snails infected) of *S. rodhaini* (top) and *S. mansoni* (bottom) at various sites in the Lake Victoria basin of Kenya over a 2 year period. Blank spaces indicate that either snails were not present or no collections were made.

Prevalence of schistosome infection varied spatially and ranged from 0.11–3.65% among positive collection sites (Table 2). Prevalence was the highest for both *S. mansoni* and *S. rodhaini* at the Car Wash site, which is an area along the shore of Lake Victoria in the city of Kisumu, Kenya, where a population of car washers earns their living by washing vehicles in the lake and is known to be infected with schistosomes [45]. *Schistosoma mansoni* was more prevalent and widespread than *S. rodhaini* which was only present at 7 of the 14 collection sites where *S. mansoni* occurred, and there were no sites where only *S. rodhaini* occurred. Total prevalence (added over time) of *S. rodhaini* was not greater than *S. mansoni* at any one site, but was more prevalent in 7 of the 169 individual collections at Nawa, Nyabera, Usare Beach, and Lwanda. Seasonal patterns of prevalence were not evident, but prevalence for both species was low between November 2004 and March 2005, and increased between September 2005 and March 2006 (Fig. 3).

### Parasite Communities Within Snails

Examination of the number of genotypes per schistosome species per infected snail included a dataset that consisted only of snails that yielded 8 or more adult worms for DNA analysis and totaled 138 snails. The total number of adults genotyped was 4,777, with a mean of 34.1 per snail (2.5 standard error), range of 8–217, and median of 24 adults per snail. Many snails were sampled over multiple days or shedding intervals that were 4–7 days apart. Snails were sampled over a mean of 2.3 (0.18 standard error) replicates, and ranged between 1 and 6 replicates. For *S. mansoni*, the 7 loci were adequate to determine that identical individuals were derived from clones and not separate sexual reproduction events. The P_sex_ values (probability that the same multilocus genotype was produced from independent sexual reproduction events) ranged from 1.2×10^−27^ to 0.000735 for this species. For *S. rodhaini*, individuals were less diverse and P_sex_ values ranged from 1.2×10^−8^ to 0.1442; however, this method does not take into account the probability that two individuals that are identical due to sexual reproduction infect the same individual snail host, which is 8.8×10^−5^ for *S. rodhaini*. Therefore, it is highly unlikely that we are missing genotypes of either species due to identical individuals in the same snail hosts.

Of the snails that yielded at least 8 adults (128 for *S. mansoni* and 12 for *S. rodhaini*, with 2 of these snails coinfected with both species), most harbored only one genotype, but multiple infections of up to 4 genotypes were found (Table 3). A total of 152 genotypes of *S. mansoni* were found in 128 infected snails and 14 genotypes of *S. rodhaini* were found in 12 infected snails. There was a significant positive correlation between prevalence and mean intensity (number of genotypes per snail) r^2^ = 0.264, p<0.05.

**Table 3:** Percentage of snails infected with 1–9 genotypes of schistosome parasites from this study and previous studies (n indicates snail sample size).

Location	Prevalence	n	1	2	3	4	5	6	7	8	9
Guadeloupe [7]	0.62	43	88.4	9.3	2.3	0	0	0	0	0	0
Brazil [43]	49–70	84	46.3	34.6	13.1	6	0	0	0	0	0
Brazil [6]	26	6	33.3	16.6	16.6	0	0	16.6	16.6	0	0
Brazil [6]	11.4	8	50	12.5	0	0	12.5	12.5	0	0	12.5
Mali [59]	3.3	35	74.2	22.9	2.9	0	0	0	0	0	0
Kenya SM	0.92	128	85.2	11.7	2.3	0.8	0	0	0	0	0
Kenya SR	0.11	12	91.7	0	8.3	0	0	0	0	0	0

Kenya SM refers to *S. mansoni* and Kenya SR to *S. rodhaini* from this study.

Three snails harbored genotypes of both *S. rodhaini* and *S. mansoni*, and were found at different sites during the last week of October of 2005 or 2006: Asembo Bay, Nyabera, and Asao. Statistical comparison with null communities indicated that the schistosome communities were structured and coinfections were more common than expected by chance at all three collecting sites, Nyabera (χ^2^ = 49.3, P<<<0.0001), Asao (χ^2^ = 140.1, P<<<0.0001), and Asembo Bay (χ^2^ = 305.4, P<<<0.0001). According to the calculated expected values, one would have to collect 15,692, 40,571, and 94,769 snails at each site, respectively, to find one coinfected snail.

### Cercarial Emergence

**Figure 4:**
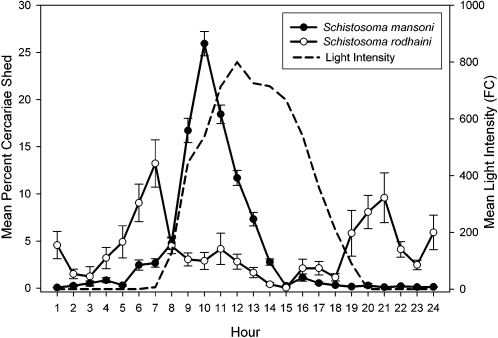
Circadian emergence of *Schistosoma mansoni* and *Schistosoma rodhaini* cercariae from naturally infected snails from the Lake Victoria region of Kenya. The mean and standard error are given based on 226 emergence profiles of *S. mansoni* and 27 for *S. rodhaini*. Light intensity is represented by the mean of all trials. Time units are hourly units beginning with 0:00–1:00 hours.

Circadian cercarial emergence profiles were generated based on 226 replicates from 100 snails infected with *S. mansoni* and 27 replicates from 8 snails infected with *S. rodhaini* (identified based on mtDNA sequences and microsatellite genotypes). Peak cercarial emergence of *S. mansoni* occurs between 8:00–13:00 and emergence of *S. rodhaini* was bimodal with a peak occurring between 5:00 and 8:00 and also 19:00 to 22:00 (Fig. 4). The ANCOVA revealed a significant interaction between parasite species and snail size, making the other effects difficult to interpret because of the uneven slopes (species: F_1,127_ = 4.702 p = 0.032; size: F_1,127_ = 0.401 p = 0.528, interaction: F_1,127_ = 5.087 p = 0.026). Using separate regressions, *S. mansoni* cercarial abundance has a significant positive relationship with snail size (F_1,117_ = 9.275 p = 0.003 r^2^ = 0.073), and *S. rodhaini* does not (F_1,10_ = 2.003 p = 0.187), a result that could be an effect of sample size since there were far fewer snails infected with *S. rodhaini.* A T-test indicated that there was no difference in cercarial production by species (T_df = 19_ = 1.237, p = 0.231).

Snails that were infected with multiple genotypes differed in the ratios of each genotype released and the proportions of each ranged from 50% of each to 95% and 5% of each. A total of 11 snails were examined for independence between the genotypes released and replicates performed over time (typically a week apart). Results indicated significant differences or non-independence between genotype and replicate for 6 of the snails (Table 4). The patterns of five of the six indicated a replacement pattern in which an initially dominant genotype is less represented in later replicates. The remaining snail showed variable proportions over 3 replicates; however, one genotype was always dominant. The five snails with nonsignificant values displayed a more constant pattern of cercarial release in which the proportions of each genotype did not change over time.

**Table 4:** Results of the Fisher's Exact tests to determine if the proportions of genotypes of *S. mansoni* released from snails are consistent over timed replicates.

Snail	P	Mode	n	R	G
Sandharvester 1	0.2076	Constant	26	3	2
Car Wash 1	0.4201	Constant	36	2	2
Asembo Bay 1	0.5120	Constant	41	2	2
Nawa 1	0.5167	Constant	48	4	2
Asao 1	0.9999	Constant	25	2	3
Car Wash 2	0.0001	Replacement	19	2	2
Car Wash 3	0.0002	Replacement	22	3	2
Homa Bay 1	0.0002	Replacement	123	2	3
Asembo Bay 2	0.0074	Replacement	119	4	2
Car Wash 4	0.0492	Replacement	18	2	4
Tilapia Beach 1	0.0001	Variable, one dominant	128	3	2

P indicates the probability of significance, n indicates the number of worms sampled, R is the number of replicates tested, and G is the number of genotypes present in a snail. Mode indicates whether the proportions remained constant over replicates, followed a replacement pattern, or was variable.

**Figure 5 pntd-0000222-g005:**
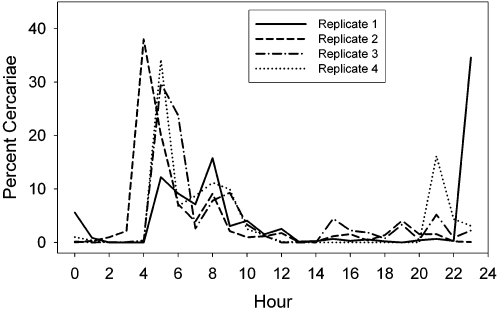
Four replicates of circadian emergence of cercariae from a snail infected with 3 genotypes of *S. rodhaini* and 1 genotype of *S. mansoni*. Time units are hourly units beginning with 0:00–1:00 hours.

**Figure 6:**
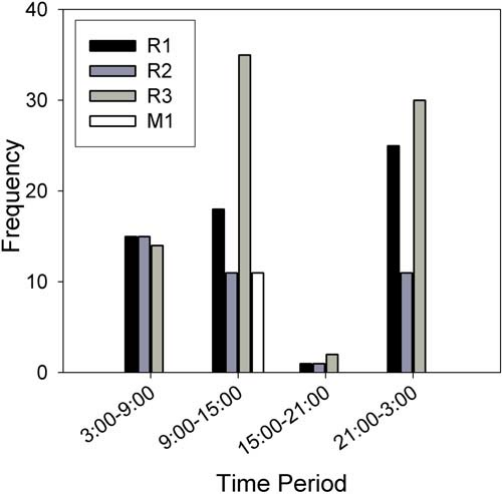
Total adults recovered of 3 genotypes of *S. rodhaini* (R1-R3) and 1 genotype of *S. mansoni* (M1) that emerged from the same snail during 4 time intervals.

For the mixed species infections, limited data were obtained from two of the three snails. For the Asao snail, only 7 worms of 2 female genotypes was recovered, 6 of which were *S. mansoni* and 1 was *S. rodhaini*. Interestingly, a single species infection of *S. rodhaini* was never found at this site. For the Asembo Bay snail, cercariae were collected twice, 28 days apart. In the first collection, 16 adults were genotyped and all were one female genotype of *S. mansoni*. Unfortunately for the second collection, only 3 adults were recovered: one was a male *S. rodhaini* and 2 were a male genotype *S. mansoni*, but a different genotype than released previously. More extensive data was obtained from the Nyabera snail, which shed 1 male genotype of *S. mansoni* and 3 genotypes of *S. rodhaini*, one male and two females. Each of 4 replicates of circadian cercarial emergence showed a peak from 8:00–10:00 hours, which corresponds to *S. mansoni* emergence, and also an earlier morning peak that corresponds to *S. rodhaini* emergence. Two replicates also showed nocturnal peaks that also correspond to *S. rodhaini* (Fig. 5). The number of adults obtained from infections of mice with cercariae collected from different time pools of these circadian profiles indicated that *S. rodhaini* was more common: 94% were of this species, and 43% of these were of the male genotype. The three-way contingency table analysis indicated that all variables, genotype (G), replicate (R), and time of day (T) and their interactions, were significant (G by R: G^2^ = 17.3, p = 0.0002; G by T G^2^ = 22.44, p = 0.0002; R by T G^2^ = 18.84, p<0.0001; G by T by R: G^2^ = 55.12, p<0.0001). The three largest standardized deviates by more than a value of 1 included the comparison of the *S. mansoni* genotype between 9:00 and 15:00 hours (3.256), a female *S. rodhaini* genotype during 3:00 to 9:00 hours (2.111), and the *S. mansoni* genotype between 21:00–3:00 hours (−2.036). These values indicate that the *S. mansoni* genotype was more common than expected during 9:00 and 15:00, the peak emergence time for this species (and the only time period that this species was collected) and less common than expected during 21:00–3:00, a time period when this species rarely emerges (Figs. 4–6). Also, one of the female genotypes of *S. rodhaini* (R3) was more common than expected during the 3:00–9:00 time period, one of the peak emergence times of this species (Fig. 6).

## Discussion

*Schistosoma mansoni* and *S. rodhaini* both have spatially and temporally patchy distributions in snails in the Lake Victoria region of Kenya and active infections (those producing cercariae) are characterized by low prevalence of about 1% combined. Although this number may be characterized as low in a relative sense, given the prodigious number of snails supported by Lake Victoria and its environs, this level of infection in snails is responsible for relatively high levels of infection in humans around the lake that can reach up to 80% in school children [46]. Most of the snails were infected with *S. mansoni*, which was about 8 times more common and more widespread than *S. rodhaini*. At every site where *S. rodhaini* was collected, *S. mansoni* was also collected, but *S. mansoni* was the sole species collected at 7 of the 14 sites. Also, *S. rodhaini* was not collected during a large part of the entire sampling period, while *S. mansoni* was present at some sites during all collection periods. The difference in the abundance and distribution of the species likely is due to differential definitive host use. *Schistosoma mansoni* primarily infects humans, which generally have larger, less subdivided, and more widespread populations than do rodents, the putative definitive hosts for *S. rodhaini*. Also, humans, and therefore their worms, are much longer lived than rodents and their worms, and serve as a more stable reservoir that continuously passes eggs and maintains the population. This difference is also reflected in the patterns of genetic diversity in that *S. rodhaini* showed little variation relative to *S. mansoni*, even when sample sizes are taken into account, reflecting a small population size for *S. rodhaini* that potentially has been bottlenecked in the past. Although *S. mansoni* outnumbered *S. rodhaini* in terms of numbers of infected snails, there was no difference in the number of cercariae produced by either species per infected snail and this number was not influenced by snail size.

Temporal patterns of prevalence were not obvious in the data, but prevalence varied spatially from 0.11–3.65% at positive sites, with the highest levels of infection occurring at Car Wash site. Although snails are in relative low abundance here due to the less than optimal habitat due to the clearing of vegetation for washing cars, human activity and fecal material are abundant so that the snails that are there are likely to be infected, including with multiple genotypes: 8 of the 21 snails with multiple infections were collected at this site. We also collected at two additional sites that were approximately 210 m and 585 m along the shore from the Car Wash site, Tilapia Beach and Powerhouse. Infection prevalence declined the further the sites were from the Car Wash site, even though snails are much more common at these sites.

Both species were overdispersed in their snail hosts, a pattern that is typical for schistosome populations in snails when prevalence is low [43]. One of the factors that likely leads to the observed pattern is the aggregation of miracidia in microhabitats occupied by particular snails [47] and low probability of contact between miracidia and snails since infection is relatively rare in this system. The fact that mean intensity and prevalence are positively correlated also suggests that probability of encounter plays a large role in determining parasite distribution, or in other words, some snails are “unlucky” and happen to be in the microhabitat where feces are deposited and eggs are hatching. Excess of multiple infections can also be explained by variability in susceptibility of infection of individual snails. Some individuals may be more susceptible or “worm-prone” and are thus likely to acquire multiple genotypes, while other snails are resistant and acquire none. Also, acquired susceptibility of snails could also lead to an excess of multiple infections. In this case, a snail that acquires one genotype becomes more susceptible to additional infections. On the other hand, lack of multiple infections can be explained by probability of encounter, differential compatibility between hosts and parasites, acquired resistance, and competition [8], [48]–[50]. One potential limitation of the methodology used in this study is the possibility of underestimating the number of genotypes that infect a snail. If rare genotypes occur in the sample (in which case they would be difficult to detect by *any* method) or if certain genotypes are rare due to low infectivity to mice, they may not be detected using our methodology. However, with a minimum sample of 16 worms, and a mean of 34.1 worms sampled per snail, this error likely is low.

The schistosome populations are structured in a way that leads to snail co-species infection more commonly than expected by random infection. Interestingly, two of these snails were also infected with multiple genotypes of one of the species so that the three snails harbored 2, 3, or 4 total genotypes. This result could be explained by the unlucky snail hypothesis mentioned above since microhabitats that are hotspots of transmission for one species could also be a hotspot for the other species. The “Worm-Prone” and the “Acquired Susceptibility” hypotheses mentioned above could also explain this pattern, but would require interspecific facilitation, a phenomenon not unknown in trematode-snail interactions [48]. Experimental infections of snails with one or both species are underway to distinguish among these possibilities. It is also possible that coinfections of definitive hosts play an important role in determining community structure at the snail level because the progeny of both species would be deposited together in the same microhabitat. Our preliminary data from worm burdens of rodents in the region have revealed only one individual that was infected with *S. rodhaini,* and that individual also was infected with *S. mansoni*.

Circadian cercarial release cycles were strongly tied to the light/dark cycle in that *S. mansoni* began to emerge as light intensity increased with the start of the daylight period, and *S. rodhaini* emerged immediately before and after the daylight period. Peak cercarial emergence of *S. mansoni* occurred earlier in the 24 hour cycle than most previously studied populations that typically undergo peak emergence when light intensity is the greatest, around noon or later, although this characteristic is known to vary among populations [29],[51],[52]. The bimodal cercarial release pattern of *S. rodhaini* has not been reported previously, and only twilight emergence was reported from populations from Burundi and Uganda [27],[29]. A possible morning peak of emergence in a Ugandan isolate of *S. rodhaini* was reported by Fripp [53]; however, his results are unclear because the snails were not monitored over a 24 hour period. In the present study, emergence of *S. rodhaini* varied among individuals and among replicates of individuals in the number of emergence peaks that occurred. In some cases both peaks occurred, but in others, only one peak occurred. Intraspecific differences in emergence time may correspond to differential definitive host use as this characteristic is likely selected for by the time that definitive hosts are present in the water and available for transmission [17],[54]. Therefore, we suspect that in Kenya *S. rodhaini* infects a host or group of hosts that are most active in the water just after sunset and right before sunrise.

Three snails were coinfected with both *S. mansoni* and *S. rodhaini*, and data from the cercarial emergence profiles of one of these snails indicate that the presence of each species does not influence the other's cercarial release patterns, which is consistent with results from other studies that have examined snails infected with both *S. haematobium* and *S. bovis* [55] or with different populations or “strains” of *S. mansoni* [56]. However, the data from the adults obtained from infections with mice also suggest that *S. mansoni* emergence is not influenced by coinfection, but *S. rodhaini* emergence may be because more adults of one genotype of this species were obtained from mice infected with cercariae that emerged between 9:00 and 15:00 hours than adults of *S. mansoni*. This result is unexpected since this is not the typical emergence time for *S. rodhaini*. Also, it is anticipated that mechanisms that separate the temporal emergence of each species would evolve particularly if they coinfect the same individual snail host because cercariae released concurrently are likely to infect the same definitive host individuals, thus potentially leading to hybridization. An alternative explanation to the observed results is that the actual number of adults of each species may be biased due to infection success since *S. rodhaini* may be better adapted to rodents, which are their presumed principal definitive hosts in nature. However, even if the proportions are biased, the data still indicate that the two species are emerging from snails concurrently.

The proportions of genotypes that emerged from snails infected with multiple genotypes varied among circadian cercarial emergence replicates (typically 1 week apart) for about half of the snails examined. Replacement of one predominant genotype by another was the most common pattern detected. It is hypothesized that infection of these snails by the different genotypes occurred sequentially with a large time interval between infections so that one genotype has developed and produces cercariae before the other has developed to the same stage. Possible complete replacement of genotypes was only detected in two snails, but was confounded by small sample sizes of worms and not included in the statistical analyses. An alternative explanation is that since cercarial production occurs in cohorts [57], the genotypes are producing their cohorts asynchronously leading to a pattern that appears to be replacement particularly when only 2 replicates of data are collected. However, in all 7 of the snails where 3 or more replicates were performed, the genotype in majority did not alternate and instead followed a pattern of replacement. The alternative to a replacement pattern was a constant pattern in which the proportions of genotypes did not differ among replicates. This constant pattern may be indicative of infections that were acquired simultaneously and are therefore at the same stage of development within the snail. Interestingly, within these infections the proportions of genotypes were mostly skewed, with the most even ratio being 61:38. This skew suggests that there are other mechanisms besides timing of infection that affect cercarial output possibly including competition between genotypes or variation in compatibility of snail and schistosome genotypes that directly affects cercarial production. These mechanisms are best addressed experimentally to determine the roles of infection timing and competition on genotype “success”, and can be performed to remove the effect of infection bias that may occur when the cercariae are introduced into mice.

Among the factors examined, this study revealed no evidence for ecologically induced isolating mechanisms that prevent *S. mansoni* and *S. rodhaini* from encountering one another and hybridizing. These species overlap on a microgeographic scale (individual sites and individual snails) and also temporally both on a seasonal scale and a circadian scale. Even though the emergence peaks of the cercariae do not directly overlap, the cercariae of these two species certainly overlap to some degree since the cercariae remain in the water column and infective for up to 9 hours, and therefore it is difficult to imagine how this would effectively isolate the two species. Also, competition within or among individual snail hosts does not seem to play a large role since coinfections were more common than expected by random infection. If anything, this observation in conjunction with the fact that *S. rodhaini* was only found in habitats also occupied by *S. mansoni*, suggests a pattern of co-occurrence as opposed to isolation. The number of cercariae produced per individual snail did not differ between the species; however, if both species share the same host pools, and if there are no strong mating barriers, it is surprising that *S. mansoni* has not driven *S. rodhaini* to extinction through hybridization since snails infected with the former species are eight times more common. However, it is possible that our sampling area represents the edge of the range of *S. rodhaini* and sampling throughout the Rift Valley may reveal larger, more stable populations that disperse to less ideal habitats through movement of snail or mammal hosts. However, the lack of genetic diversity suggests that migration from larger populations is not occurring on a regular basis.

It is unknown how long *S. mansoni* and *S. rodhaini* have been in contact in Kenya and if their original divergence was due to sympatric or allopatric speciation. If the latter has occurred and we are witnessing relatively recent secondary contact, then this situation seemingly parallels one occurring in Cameroon in which *S. intercalatum* is thought to be endangered due to its interactions with *S. haematobium* and *S. mansoni* [58]. Decline of *S. intercalatum* has occurred in recent years (1968-present) and is directly correlated with the introduction of *S. haematobium* in the region [58]. However, the molecular data suggest *S. mansoni* and *S. rodhaini* diverged approximately 2.8 million years ago [3], and it seems likely that they have coexisted in the Lake Victoria basin for a long time. The most likely isolating mechanism separating the two species is the difficulty of *S. rodhaini* in infecting non-human primates [15] and presumably humans as well, and the preponderance of *S. mansoni* infections in humans. We have collected both species in the same rodent hosts (unpublished observations) but the relative frequency with which such coinfections occur may be insufficient to break down the genetic differences between the two species, or mate recognition systems may hinder interspecific reproduction when they do encounter each other in a host. What is still lacking is a full understanding of the definitive hosts used by *S. rodhaini* to propagate itself, whether these hosts are routinely colonized by *S. mansoni*, and whether the species will hybridize if they encounter each other in the same host. Future monitoring of schistosome populations in Western Kenya and further studies on introgressive hybridization will give further insight on the interactions between these species.
